# Role of Post-Translational Modifications in Colorectal Cancer Metastasis

**DOI:** 10.3390/cancers16030652

**Published:** 2024-02-03

**Authors:** Na Peng, Jingwei Liu, Shuangshuang Hai, Yihong Liu, Haibo Zhao, Weixin Liu

**Affiliations:** 1 Department of Gastroenterology, The First Affiliated Hospital of China Medical University, Shenyang 110001, China; 2021120637@cmu.edu.cn (N.P.); sshai@cmu.edu.cn (S.H.); ygliu@cmu.edu.cn (Y.L.); 2022120660@cmu.edu.cn (H.Z.); 2 Department of Anus and Intestine Surgery, The First Affiliated Hospital of China Medical University, Shenyang 110001, China; jwliu@cmu.edu.cn

**Keywords:** colorectal cancer, metastasis, post-translational modifications, therapy

## Abstract

**Simple Summary:**

The presence or absence of colorectal cancer metastasis largely determines the survival time of patients. Post-translational modifications (PTMs) have been reported to regulate various cellular biological processes. But how they influence CRC remain to be comprehensively summarized. PTMs exhibit diverse effects on CRC metastasis, which can be either protective or detrimental. This depending on the complex network formed by different PTMs their regulatory signaling pathways. Here, we discuss the PTMs involved in CRC metastasis to help researchers to understand the research progress of PTMs related to colorectal cancer metastasis. Targeting PTMs implicated in colorectal cancer metastasis might be a promising approach in the clinical treatment in the future.

**Abstract:**

Colorectal cancer (CRC) is one of the most common malignant tumors of the digestive tract. CRC metastasis is a multi-step process with various factors involved, including genetic and epigenetic regulations, which turn out to be a serious threat to CRC patients. Post-translational modifications (PTMs) of proteins involve the addition of chemical groups, sugars, or proteins to specific residues, which fine-tunes a protein’s stability, localization, or interactions to orchestrate complicated biological processes. An increasing number of recent studies suggest that dysregulation of PTMs, such as phosphorylation, ubiquitination, and glycosylation, play pivotal roles in the CRC metastasis cascade. Here, we summarized recent advances in the role of post-translational modifications in diverse aspects of CRC metastasis and its detailed molecular mechanisms. Moreover, advances in drugs targeting PTMs and their cooperation with other anti-cancer drugs, which might provide novel targets for CRC treatment and improve therapeutic efficacy, were also discussed.

## 1. Introduction

**Figure 1:**
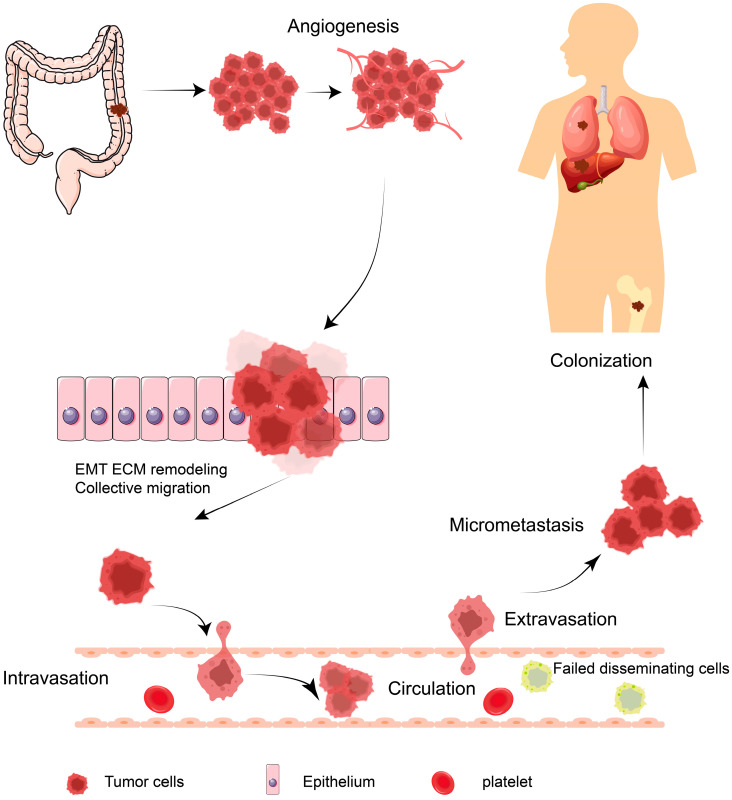
Schematic representation of the crucial mechanisms of CRC metastasis. The key steps of CRC cell metastasis from the primary lesion to other metastatic sites. The arrow represents the next stage of biology.

Colorectal cancer (CRC) is a common malignant tumor of the digestive tract and a leading cause of death worldwide [1], characterized by a high incidence and poor prognosis. Although diagnostic technology has advanced and endoscopic screening is widely used, the mortality of CRC is worryingly high. The main reason is that CRC is often diagnosed with tumor metastasis. The rate of patient recurrence after radical resection of the primary tumor is approximately 30–40%, while the 5-year survival rate is only 50–60% [2,3]. CRC metastases mainly include direct extension, vascular/lymphatic spread, portal venous spread, and peritoneal dissemination. Common sites of metastases from colorectal cancer include the adjacent lymph node, liver, lung, peritoneum, and bone [4,5], of which the most common metastatic site of CRC in patients is the liver [4]. CRC metastasis is a complex, multi-step biological process that includes the proteolysis of the extracellular matrix, alteration of adhesions, local infiltration, angiogenesis, intravascular dissemination, immune escape, distant formation of emboli, and survival in new environments [6] (Figure 1). Elucidating the molecular mechanisms of CRC metastasis would greatly benefit the treatment and prognosis of CRC patients. 

Post-translational modifications (PTMs) are processes that take place on a protein either shortly after its translation by ribosomes or after its folding and localization are complete. These modifications are usually catalyzed by enzymes and involve the addition of chemical groups, sugars, or proteins to specific residues of the target protein [7]. Various types of PTMs are identified in humans, including, but not limited to, phosphorylation, ubiquitination, SUMOylation, acetylation, methylation, fatty acylation, glycosylation, and neddylation [8]. PTMs significantly change protein function by fine-tuning protein stability, localization, or interactions, which helps to orchestrate complicated processes in response to intracellular and extracellular environmental changes [9]. It has been reported that PTMs regulate various biological functions such as cell cycle progression, differentiation, survival, proliferation, signal transduction, and transcriptional regulation. Emerging evidence suggests that PTMs constitute a central mechanism, alongside the genetic code, to regulate complex cellular activities. 

It is suggested that cancer-related molecular alterations are not only caused by variations among DNA or mRNA/protein expression but are also the result of the complex reprogramming of signaling pathways mediated by the PTM of various proteins [10]. In recent years, an increasing number of studies have suggested that the dysregulation of PTMs is closely implicated in various aspects of malignancies [11]. Importantly, PTMs play pivotal roles in the key steps of colorectal cancer metastasis, including avoiding immune surveillance, intravasation into the vasculature, extravasation from the vasculature, enabling replicative immortality, and inducing angiogenesis [12,13,14]. For example, the glycation modification at the N192 and N200 sites of PD-L1 contributes to tumor cells’ escape from T-cell recognition and, subsequently, CRC metastasis [15]. In addition, DOT1L acetylation in CRC tissues is not only significantly higher than in corresponding adjacent normal tissues but also regulates CRC migration, invasion, and metastasis [16]. Another study demonstrated that phosphorylated vimentin expression levels in primary tumors are significantly lower than in metastatic tumors in CRC patients [17]. A study based on proteomics and bioinformatics documented that global protein ubiquitination between human primary and metastatic colon adenocarcinoma tissues is notably different, and ubiquitination of CDK1 may be a potential mechanism of CRC metastasis [18]. Here, we comprehensively summarized multiple post-translational modifications that participate in colorectal cancer metastasis and their detailed molecular mechanisms. It is anticipated that targeting specific modifications implicated in CRC metastasis could improve clinical treatment and survival for patients with colorectal cancer. 

## 2. Phosphorylation and CRC Metastasis

**Figure 2:**
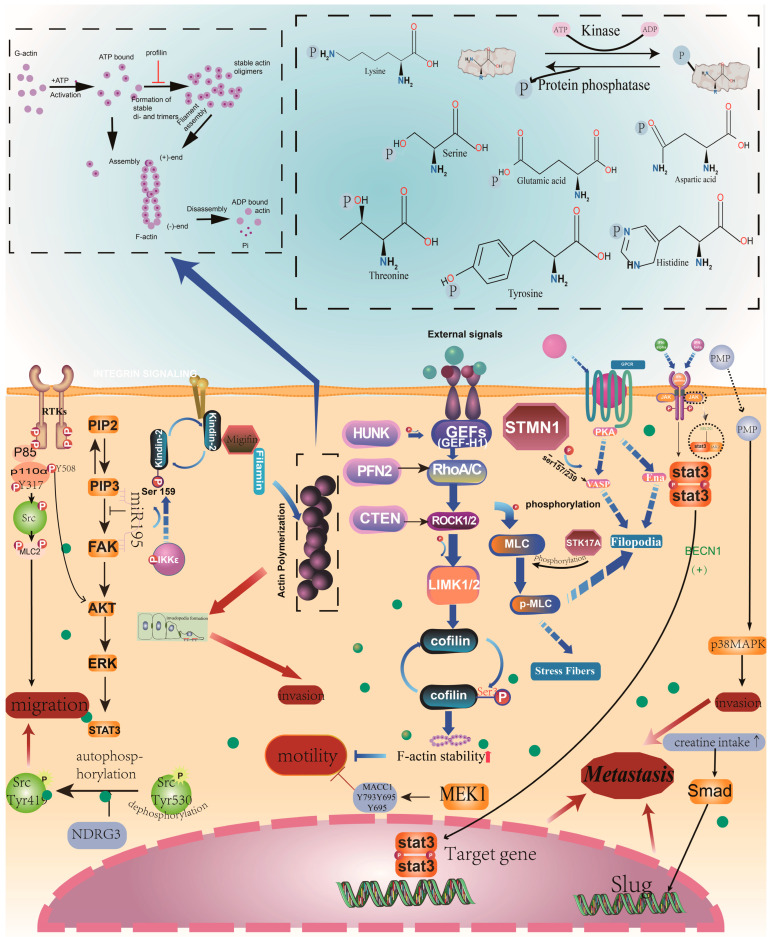
Phosphorylation regulates the invasion and migration of CRC cells. The activation of invasion and the increase of migration ability is partly triggered by phosphorylation. The red arrow promotes metastasis, and the red vertical T font inhibits metastasis.

Phosphorylation is the most abundant PTM of proteins [19]. Protein kinases are responsible for adding phosphate groups at the serine/threonine/tyrosine (Ser/Thr/Tyr) residues of the target proteins to maintain the dynamic balance of intracellular phosphorylation (Figure 2). Multiple studies have suggested that aberrant phosphorylation contributed to CRC metastasis [20]. 

### 2.1. Phosphorylation Regulates Cytoskeleton Rearrangement

In the process of tumor cell invasion and migration, cytoskeleton remodeling occurs throughout the whole process, which is the executor of tumor cell invasion and migration [21]. Therefore, understanding and exploiting the role of phosphorylation in actin cytoskeleton remodeling will help to understand the invasion and metastasis of CRC.

It has been reported that IKKε promotes metastasis in CRC by phosphorylating kindlin-2 at S159, which induces invadopodia formation and governs its activity [22]. STK17A has been suggested to modulate metastasis of CRC by phosphorylating MLC [23], resulting in alterations in cell size and induced widespread membrane blebbing, a phenotype often associated with the activation of cell contractility. RhoA-LIMK-cofilin1 signaling regulates CRC metastasis because the phosphorylation at Ser3 in cofilin can enhance the stability of F-actin and alter cell migration ability [24]. STMN1, a microtubule destabilizing protein overexpressed in CRC, was suggested to induce microtubule instability and promote CRC metastasis. The underlying mechanism is that STMN1 regulates VASP phosphorylation, altering actin polymerization, cell motility, and spindle fiber stability [25]. In addition, PFN2 serves a suppressive role in the metastasis of CRC through myosin light chain phosphorylation to regulate cytoskeletal rearrangements [26]. Src is a kinase that consists of focal adhesions, and Cten-Src signaling was found to promote cell migration, cell invasion, and CRC metastasis [27]. Additionally, HUNK phosphorylates GEF-H1 at serine 645, which consequently activates RhoA and further phosphorylates LIMK-1/CFL-1. By this means, the stability of F-actin is improved, and CRC metastasis is inhibited [28] (Figure 2). 

### 2.2. Phosphorylation Regulates EMT

**Figure 3:**
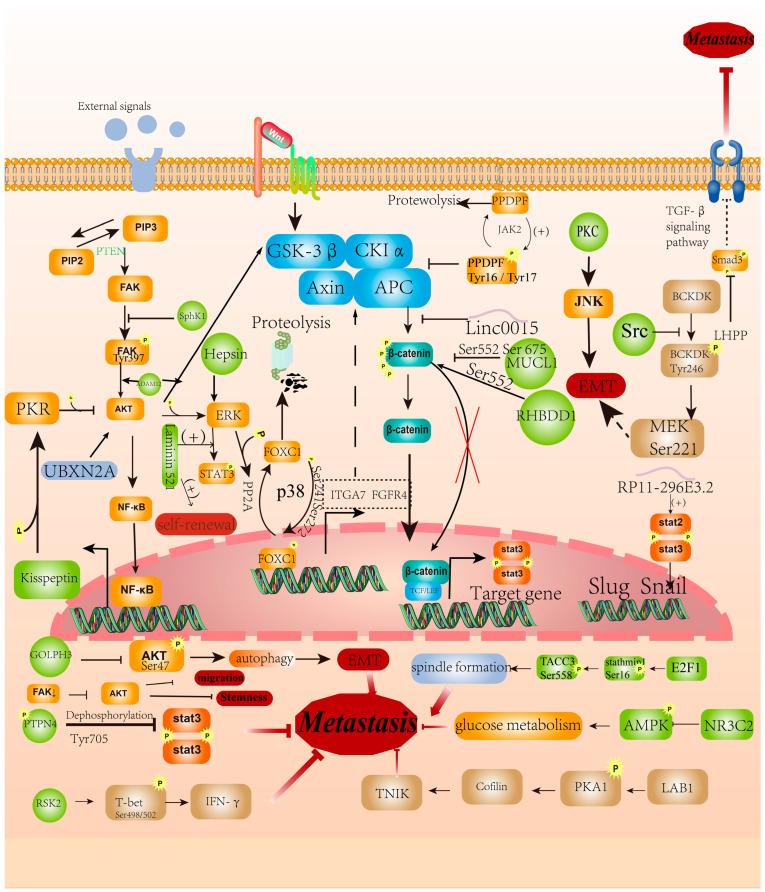
Phosphorylation regulates EMT and other functions of CRC cells. The phosphorylation signaling pathway is associated with EMT and other cellular functions, including autophagy, stemness, and glucose metabolism of CRC cells. The red arrow promotes metastasis, and the red vertical T font inhibits metastasis.

EMT is a complex pathophysiological process that plays a role in embryonic development, tissue repair, fibrosis, and cancer progression [29]. The protein kinase C (PKC) family is a group of evolutionarily-conserved serine/threonine kinases that facilitate JNK phosphorylation, as well as EMT, and are over-expressed in the metastatic tissues of CRC [30]. SphK1 concatenates the FAK/AKT/MMPs axis with its phosphorylation function, promoting the migration and metastasis of colon cancer [31]. Src phosphorylates BCKDK at the tyrosine 246 site, which decreases E-cadherin while increasing N-cadherin and Vimentin, thus leading to the migration and invasion of CRC cells [32]. As a protein tyrosine phosphatase, PTPN4 directly interacts with pSTAT3 and dephosphorylates it at the Tyr705 residue, which can suppress rectal cancer metastasis [33]. A rhomboid domain containing 1 (RHBDD1) is an intramembrane serine protease that contributes to CRC cell metastasis in vivo and in vitro. Mechanically, RHBDD1 influences Wnt signaling pathway activity by regulating the phosphorylation of ser552 and ser675 of β-catenin [34]. Ribosomal S6 kinase 2 (RSK2) is a serine/threonine kinase that can regulate gene expression by phosphorylating transcription factors. RSK2 has been found to induce the phosphorylation of T-bet at serines 498 and 502, resulting in the inhibition of colon cancer metastasis [35]. In addition, the knockout of LKB1 promotes CRC metastasis because LKB1 can directly phosphorylate PAK1 and inhibit cell motility. ADAM12 promotes EMT and CRC metastasis via activating the PI3K/PDK1/AKT and PI3K/AKT/GSK-3β signaling pathways [36]. The transcription factor FOXC1, phosphorylated by P38 at Ser241 and Ser272 [37], can directly transactivate ITGA7 and FGFR4 to promote CRC metastasis [38]. MUCL1 is suggested to regulate cell invasion and migration by inhibiting EMT in CRC by activating β-catenin via its Ser-552 phosphorylation and nuclear accumulation [39]. LHPP has been found to inhibit CRC metastasis by repressing Smad3 phosphorylation to inhibit the TGF-β signaling pathway [40]. Kisspeptin-mediated phosphorylation of PKR inhibits the phosphorylation of both AKT and ERK by increasing PP2A activity. Therefore, kisspeptin treatment can block the migration of CRC cells [41]. Hepsin is a Type II transmembrane serine protease that is involved in CRC invasion and metastasis via increasing Erk1/2 and STAT3 phosphorylation [42]. Neogenin inhibits CRC progression and metastasis by binding to Merlin and subsequently enhances YAP phosphorylation [43]. UBXN2A inhibits CRC growth and metastasis by reducing AKT phosphorylation downstream of the dominant oncogenic pathway in CRC [44]. As for non-coding RNAs, linc00152 can block CK1-mediated cytoplasmic β-catenin phosphorylation and consequently facilitate β-catenin nuclear translocation, thus promoting colon cancer EMT and metastasis [45]. lncRNA RP11-296E3.2 serves as a molecular chaperone to activate the transcription and phosphorylation of STAT3, which promotes CRC metastasis [46] (Figure 3).

### 2.3. Phosphorylation Regulates Invasion and Migration

The uptake of creatine has been suggested to significantly enhance CRC metastasis. The underlying mechanism is that creatine drives tumor invasion and metastasis via the Smad/Snail and Slug axis, which triggers MPS1 to phosphorylate Smad2 and Smad3 [47]. BECN1 negatively regulates CRC metastasis by modulating the phosphorylation of STAT3. In particular, BECN1 can directly interact with STAT3 and JAK2, which prohibits the interaction between STAT3 and JAK2 [48]. NDRG3, an N-myc downregulated gene, has been reported to promote the migration and invasion of CRC cells by activating c-Src at Tyr419 [49]. Significantly downregulated in rectal cancer, tumor suppressor miR-195 regulates the metastasis of rectal cancer by affecting the phosphorylation of PI3K/AKT signaling [50]. Targeting MEK1 has been revealed to reduce MACC1 tyrosine phosphorylation and restrict colorectal metastasis induced by MACC1 [51]. In addition, p110α Y317 phosphorylation was found to promote CRC metastasis processes by mediating the Src-MLC2 signaling pathway [52]. It has been found that p110α Y317 phosphorylation promotes the growth and metastasis of CRC cells by regulating AKT signaling. Platelet-derived microparticles (PMPs) can act as intracellular signaling vesicles, promoting the phosphorylation of p38MAPK and enhancing the mobility and invasive properties of CRC cells [53] (Figure 2).

### 2.4. Phosphorylation Regulates Other Characteristics of CRC Cells

In addition to the above-mentioned aspects of CRC metastasis, other characteristics of CRC cells related to metastasis have also been unraveled, including autophagy, the cell cycle, metabolism, and stemness [54]. 

Golgi phosphoprotein 3 (GOLPH3) has been reported to suppress the phosphorylation of Akt at Ser473, which regulates autophagy to promote CRC metastasis [55]. In addition, the transcription factor E2F1 has been shown to facilitate cell cycle progression of CRC cells via regulating stathmin1 and TACC3 [56]. In particular, stathmin1 phosphorylation at Ser16 is necessary for the TACC3 phosphorylation at Ser558, which induces spindle formation. It has been found that the expression level of NR3C2 in CRC patients was negatively correlated with advanced-stage and distant metastasis. Mechanically, NR3C2 inhibits glucose metabolism by decreasing the expression of HK2 and LDHA, as well as the phosphorylation of AMPK [57]. Emerging evidence suggests that cancer cell stemness is another important factor for metastasis initiation. Cancer stem cells refer to a small number of tumor cells with strong self-renewal abilities and multiple differentiation abilities. Laminin 521 has been suggested to promote STAT3 phosphorylation and CRC cell self-renewal [58]. Laminin alpha 5 was detected in 7/10 liver metastases from CRC patients and in mouse liver metastasis xenografts, with the expression of metastasis cancer stem cell markers. Additionally, the inactivation and phosphorylation of AKT decreased the stemness of CRC cells. The downregulation of Focal adhesion kinase (FAK) has been shown to inhibit the stem-like properties and migration of CRC cells, as FAK alters the regulation of AKT phosphorylation [59] (Figure 3).

## 3. Ubiquitination/Deubiquitination and CRC Metastasis

**Figure 4:**
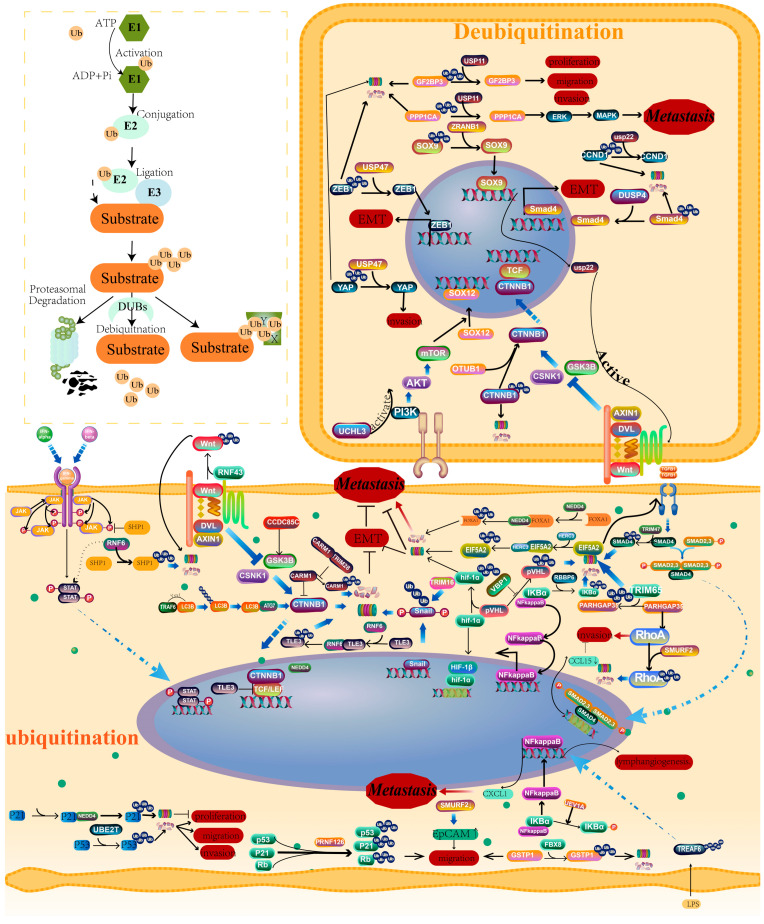
Ubiquitination and deubiquitination regulate the metastasis of CRC cells. Ubiquitination and deubiquitination lead to the degradation or increased stability of key proteins that play a role in colorectal cancer metastasis.

As one of the most prevalent posttranslational modifications, ubiquitination modification is the process of covalently binding one or more ubiquitins to a target protein using an enzymatic reaction [60]. Ubiquitin is a small protein composed of 76 amino acids, which is a highly conserved globulin in eukaryotes. The ubiquitination of proteins is governed by enzymes that catalyze ubiquitin activation (E1s), conjugation (E2s), and ligation of protein targets (E3s). The E3 ubiquitin ligase is responsible for substrate recognition and determines the specificity of the reaction [61] (Figure 4). We summarized key information from the relevant literature, including E3’s name, substrate, roles in CRC metastasis, cellular function (experimentally verified), and signaling pathway. The detailed information on a total of 13 E3 ubiquitin ligases/ligase complexes is summarized in Table 1.

### 3.1. Ubiquitination Regulates EMT

EMT transformation is a vital process of CRC metastasis, and targeting this process might exert a regulatory role in metastasis. It has been reported that neural precursor cell-expressed developmentally downregulated protein 4 (NEDD4) binds to FOXA1, which mediates the ubiquitination and degradation of FOXA1 and contributes to the metastasis of xenograft tumors. The silencing of NEDD4 inhibited the viability, invasion, and epithelial-to-mesenchymal transition of CRC cells [62]. HERC3 promotes the ubiquitination and degradation of EIF5A2, which inhibits the metastasis of CRC. In addition, HERC3 inhibits CRC cell metastasis through EIF5A2/TGF-/Smad2/3 signals [64]. TRIM16 promoted the ubiquitination and degradation of Snail, thus suppressing CRC cell metastasis by regulating EMT [73]. TRAF6 can drive selective autophagic CTNNB1 degradation machinery to curb EMT and CRC metastasis. Wu et al. revealed the underlying mechanism by which TRAF6 uses its LC3-interacting region, ‘YxxL,’ to interact with MAP1LC3B/LC3B and catalyze the K63-linked polyubiquitination of LC3B. Mechanistically, this facilitates the recognition of CTNNB1 by LC3B for selective autophagic degradation. Thus, CTNNB1 degradation can effectively suppress EMT and CRC metastasis [75]. 

The ring finger protein 6 (RNF6) is a member of the E3 ubiquitin ligase family, which has a RING-finger domain at the C-terminus that can contribute to its E3 ubiquitin ligase activity. RNF6 can stabilize STAT3 phosphorylation by ubiquitylating and degrading SHP-1, which ultimately mediates the JAK/STAT3 pathway activation in CRC cells. The downregulation of RNF6 significantly suppresses CRC cell growth and metastasis [65]. In addition, the RNF6-mediated degradation of TLE3 promotes EMT and CRC metastasis [66]. RBBP6, a RING finger-domain E3 ubiquitin ligase, has been shown to be positively correlated with CRC patients’ distant metastases. Mechanistically, RBBP6 promotes the activation of the NF-κB signaling pathway through ubiquitination and degradation of the NF-κB inhibitor IκBα [76]. Hypoxia is one of the leading causes of EMT and metastasis initiation, which makes it a common characteristic of CRC. VBP1 stabilizes pVHL by repressing pVHL ubiquitination, which regulates HIF-1α-induced EMT and CRC metastasis by stabilizing pVHL [69]. CCDC85C overexpression promoted β-catenin phosphorylation and ubiquitination by enhancing the functionality of GSK-3β, which thereby serves as a suppressor of CRC cell proliferation and migration [81] (Figure 4).

### 3.2. Ubiquitination Regulates Invasion and Migration

NEDD4 has been found to mediate the ubiquitination of p21 and promote CRC invasion [63]. TRIM47 promotes the ubiquitination and degradation of SMAD4 and subsequently leads to CCL15 overexpression. The CCL15-CCR1 signaling can lead to an increase in the ability of CRC cells to invade [71]. CRC patients with metastases had a higher level of TRIM65. TRIM65 correlated with ARHGAP35 and promoted the production of migration-related structures; thus, it enhanced CRC metastasis to distant organs [70]. TRIM28 can inhibit the migration and invasion of CRC in vivo and in vitro. The underlying mechanism is that TRIM28 protects CARM1 from proteasome-mediated degradation, by which WNT/β-catenin signaling can be inhibited [72]. RNF126 ubiquitinates WT p53, as well as its downstream target p21 and Rb, by activating the ubiquitin-proteasome system, which promotes cell mobility in CRC cells [67]. FBX8 regulates the ubiquitination and stability of GSTP1, which inhibits the invasion and metastasis of CRC cells in vivo and in vitro [77]. SMURF2 acts as an E3 ubiquitin ligase to mediate the ubiquitination and degradation of RhoA, thereby decelerating the migration and invasion of CRC cells [79] (Figure 4). 

### 3.3. Ubiquitination Regulates Stemness

The ring finger protein 43 (RNF43), known as a tumor suppressor, inhibits the Wnt pathway via the ubiquitination and degradation of Wnt receptors of the Frizzled (Fzd) family, located at the cell membrane. It has been reported that silencing RNF43 enhances the stemness properties of colon cancer cells [68]. A knockdown of SMURF2 can accelerate cell migration and promote the expression of EpCAM, which is a cancer stem cell marker. As Smurf2 is significantly correlated with the stem cell-like properties of colon cancer cells, Smurf2 may be a target in the progression of CRC from original tumor growth to metastatic cascade and even recurrence after the initial hepatectomy [80] (Figure 4). 

### 3.4. Ubiquitination Regulates Lymph Node Metastasis

TRAF6-mediated ubiquitination regulates the LPS-NF-κB-VEGF-C signaling pathway, which triggers the initial activation step to affect lymphangiogenesis. Importantly, the overexpression of TRAF6 promoted lymphangiogenesis, while a TRAF6 knockdown inhibited lymphangiogenesis. In particular, TRAF6 affected the migration, invasion, and lymphatic metastasis of CRC through its ubiquitination activity [74]. FBXW7, an E3 ubiquitin ligase, is a tumor suppressor that can regulate the ubiquitination and proteolysis of various targets such as cyclin E, c-Jun, c-Myc, and Notch. Clinical data indicated that FBXW7 protein expression was associated with clinicopathological parameters such as lymph node metastasis and an advanced TNM stage [78]. 

### 3.5. Ubiquitin-Conjugating E2 Enzymes and CRC Metastasis

It has been suggested that UBE2T promotes the growth, proliferation, invasion, and metastasis of CRC cells by ubiquitinating the p53 protein and regulating the expression of downstream related proteins, thereby promoting the occurrence and development of CRC [82]. UEV1 encodes a ubiquitin (Ub)-conjugating enzyme variant (Uev), which is able to promote cell migration and invasion. UEV1A overexpression can promote IκBα phosphorylation and NF-κB translocation into the nucleus, and this effect is absolutely dependent on its physical interaction with Ubc13. NF-κB, in turn, up-regulates CXCL1 expression to enhance colon cancer cell metastasis [83] (Figure 4). 

### 3.6. Deubiquitination and CRC Metastasis

DUBs are proteases that remove the ubiquitin moiety from the ubiquitinated substrate to antagonize the modification mediated by the E3 ubiquitin ligase. In recent years, many studies have found that deubiquitinating enzymes are highly expressed in CRC tissues with metastases.

As the largest family of deubiquitinating enzymes, the USP family has about 60 proteases ranging in size from 50 kDa to 300 kDa. Current studies have shown that USP is involved in the migration and invasion of CRC cells. USP11 promotes the proliferation, migration, and invasion of CRC cells by modulating IGF2BP3 stability [84]. Another study has the same point, namely, that the overexpression of USP11 promotes metastasis of CRC cells both in vitro and in vivo by activating the ERK/MAPK pathway and stabilizing PPP1CA. Therefore, USP11 might be a novel biomarker for predicting liver metastasis in CRC [85]. Sox9 is a transcription factor, playing an oncogenic role in multiple human cancers, which is associated with an enhanced epithelial–mesenchymal transition, lymph node metastasis, and stem-cell maintenance in tumors. ZRANB1 can decelerate the ubiquitination of Sox9 to stabilize it in CRC cells, which enhances activation of the downstream USP22/Wnt/β-catenin pathway to increase stem-cell-like properties and promote tumor progression [86]. CCND1 serves as a rate-limiting regulator of G1-S progression, which is identified as a USP22 deubiquitylation substrate. CCND1 is protected from proteasome-mediated degradation by USP22, and aggressive growth phenotypes in CRC cells are inhibited [87]. ZEB1 is considered to be a vital EMT-related transcription factor and is tightly related to the occurrence and development of CRC. USP43 directly interacts with and deubiquitinates ZEB1, thus affecting the cell migration and EMT of CRC [88]. In addition, the USP47-YAP signaling axis can promote CRC cell invasion by protecting YAP from degradation by proteases, increasing YAP expression [89]. As a family member of DUBs, the deubiquitinating enzyme UCHL3 has been found to facilitate CRC cell migration and invasion by regulating SOX12 via the AKT/mTOR signaling pathway [90]. OTUB1, known as a K48 linkage-specific deubiquitinating enzyme, promotes β-catenin expression by suppressing its protein degradation and regulates CRC metastasis [91]. In addition, DUSP4 plays a core role in aggravating cell migration in CRC by regulating the ubiquitylation and degradation of Smad4, a key protein controlling EMT [92] (Figure 4). 

## 4. Glycosylation and CRC Metastasis

**Figure 5:**
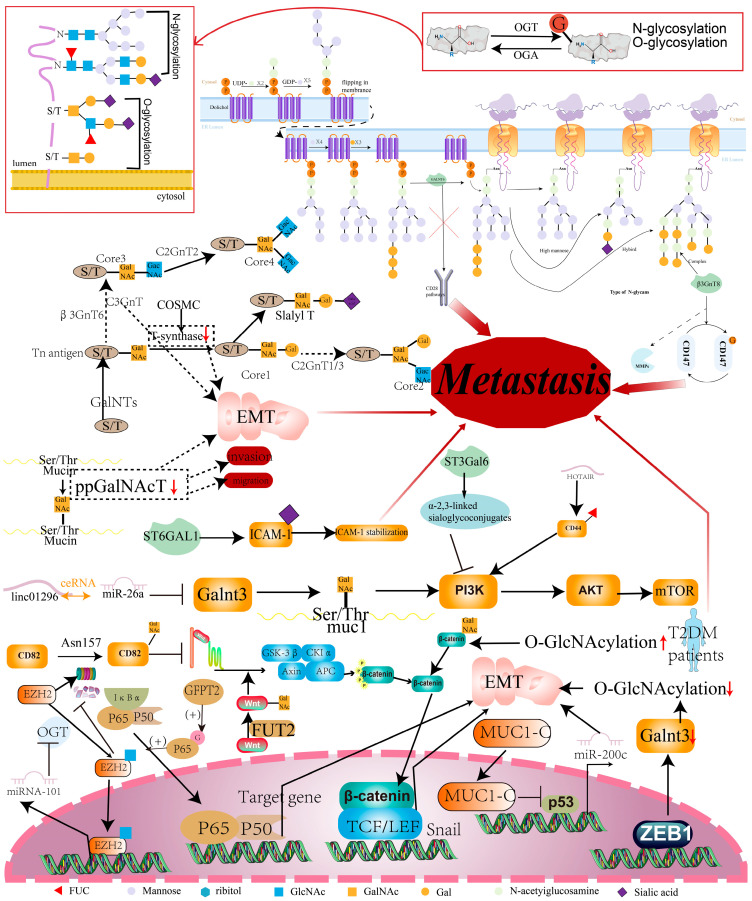
Glycosylation regulates the metastasis of CRC cells. Glycosaminyltransferase induces glycosylation to regulate multiple signaling pathways involved in CRC metastasis. The red arrow promotes metastasis.

Glycosylation is an important and highly regulated additional secondary mechanism of proteins in cells. Protein glycosylation begins in the endoplasmic reticulum and ends in the Golgi apparatus [93]. Protein glycosylation can be classified into four categories, namely, O-glycosidic bond formation with the hydroxyl groups of serine, threonine, hydroxylysine, and hydroxyproline as the connection points. The two main types of glycosylation are N- and O-glycosylation of the junctions [94] (Figure 5), which differ in the type of glycoprotein-protein junctions. Glycosylation plays a key role in determining the structure, function, and stability of proteins. 

### 4.1. Glycosaminyltransferase and CRC Metastasis

Glycosylation is performed by various glycosyltransferases, and the imbalance of enzyme expression can cause changes in the target protein or downstream pathways, which will alter the characteristics of tumor cells [95]. The polypeptide N-acetylgalactosaminyltransferase (GALNT) family participates in the initial stage of mucin O-glycosylation, which can dramatically suppress AKT, the downstream CD28 signaling pathway, and is associated with lymph node metastases of CRC [96]. β1,3-N-acetylglucosaminyltransferase (β3GnT8) is the key enzyme that catalyzes the formation of polylactosamine glycan structures by transferring GlcNAc to tetra-antennary β1-6-branched N-glycan. β3GnT8 has been found to catalyze the N-glycosylation of CD147, thus promoting CRC invasion by modulating the polylactosamines of CD147 [97]. Polypeptide N-acetylgalactosaminyltransferase 4 plays an important role in the initiation phase of the mucin-type O-glycosylation in cells. When ppGalNAc-T4 expression was down-regulated in CRC cells, the ability of cell invasiveness, migration, and EMT were promoted [98]. GalNAc transferase, involved in O-glycosylation (GALNT3), is a target of the transcription factor ZEB2; ZEB2 regulates EMT, whose expression is inversely correlated with ZEB2. The negative regulation of O-glycosylation by ZEB2 contributes to CRC metastasis, and the reduced GALNT3 levels are associated with worse survival [99]. 

T-synthase is the key enzyme required for the biosynthesis of mature O-glycans. It has been found that a knockout of T-synthase in HCT116 cells enhanced cell migration and invasiveness by triggering the molecular process of the EMT pathway [100]. Glutamine fructose-6-phosphate amidotransferase 2 (GFPT2) is the key enzyme of the hexosamine biosynthesis pathway. GFPT2 has been found to promote the glycosylation of p65 and subsequently lead to NF-κB pathway activation, thus resulting in the migration, invasion, and metastasis of CRC cells [101]. Core 3 synthase demonstrated a positive correlation with the metastatic capacity of CRC cells [102]. In addition, a novel mechanism has been provided that links the mucin-type core 3 O-glycan to the EMT–MET plasticity of CRC. The p53-miR-200c regulatory axis can be activated by MUC1-C nucleus translocation due to core 3 O-glycosylated MUC1 [103]. GALNT3 is known to modulate the O-glycosylated MUC1 and PI3K/AKT pathway. It has been reported that the linc01296/miR-26a axis regulates GALNT3 expression and further influences CRC malignant behavior such as metastasis [104]. Another study indicates that the overexpression of sialyltransferase ST6GAL1 can promote the sialylation and stability of ICAM-1, thereby inhibiting CRC metastasis [105]. The glycosyltransferase FUT2 mediates the glycosylation of Wnt2 and further activates the Wnt/β-catenin pathway to promote tumor growth and the metastasis of CRC [106] (Figure 5). 

### 4.2. Glycosylation of Key Proteins and CRC Metastasis

In addition to changes in glycosyltransferases, the glycosylation of key proteins also contributes to CRC metastasis. As one of the most overexpressed tumor antigens, the Tn antigen promotes CRC metastasis by activating the EMT pathway [107]. T-synthase in the Golgi apparatus modifies the Tn antigen to form elongated and complex O-glycans, which is a hallmark of abnormal O-glycosylation in CRC. GlcNAcylation can affect a key cellular process in the invasion and CRC metastasis of the EMT process. For instance, β-catenin transcriptional activity is elevated when the modulation of O-GlcNAcylation is elevated [108]. As a non-protein coding RNA, ST3Gal6 can mediate the migration and invasion of CRC cells by catalyzing α-2, 3 sialylation to inhibit the PI3K/Akt pathway. Clinical data indicates that ST3Gal6-AS1 levels have a negative correlation with CRC lymphatic metastases and distant metastases [109]. The fact that O-GlcNAcylation promotes metastasis in CRC may partly be explained by the fact that miR-101/O-GlcNAcylation/EZH2 signaling forms a feedback loop. The miRNA-101 targets OGT, which can transcriptionally silence miR-101 as feedback in an EZH2-dependent manner, upregulating OGT and EZH2 to promote CRC metastasis [110]. Some scholars focused on the relationship between glycosylation and the metastasis of diabetic CRC. In CRC tissues of patients with T2DM, β-catenin/Snail-mediated EMT can be promoted via the O-GlcNAcylation of β-catenin [111]. As for lncRNAs, HOTAIR increases the level of α1, 3-fucosylation of CD44 and thus affects CRC metastasis by triggering the PI3K/AKT/mTOR pathway [112]. Further, the N-glycosylation of CD82, which is a glycoprotein and belongs to the tetraspanin superfamily, at the Asn157 site can induce CRC cell mobility. In the meantime, the Wnt/β-catenin signaling pathway can be regulated and, therefore, inhibit EMT [113] (Figure 5). 

## 5. Non-Classical PTMs and CRC Metastasis

**Figure 6:**
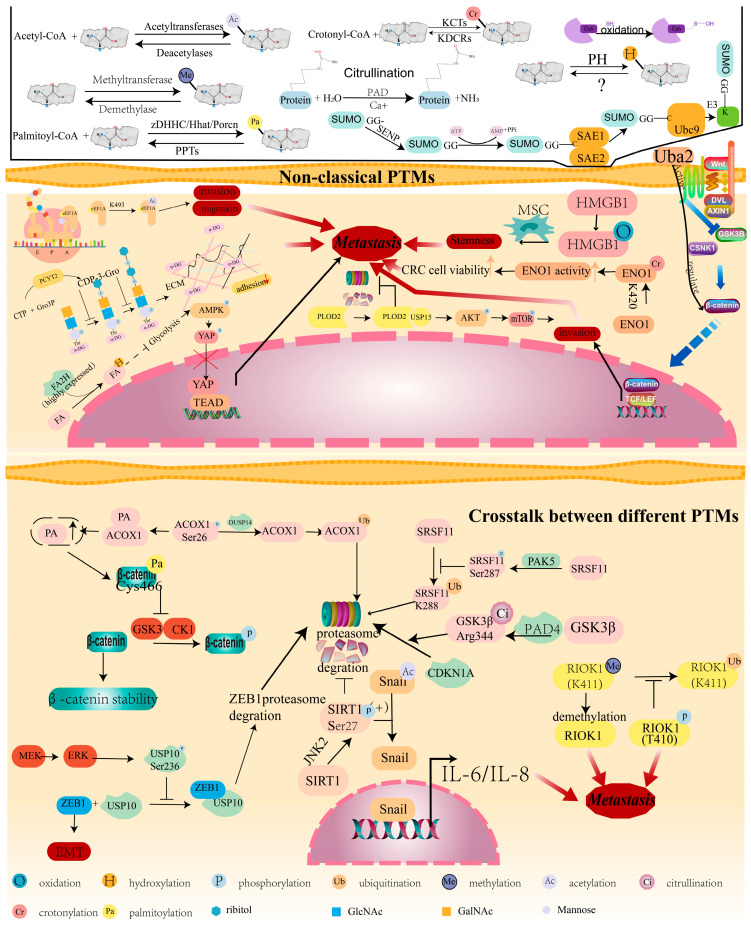
Non-classical PTMs and crosstalk between different PTMs contribute to CRC metastasis. The red arrow promotes metastasis.

In addition to the classical modifications, there are also many non-classical post-translational modifications such as succinylation, acetylation, hydroxylation, and crotonylation. For instance, glycerol phosphate modification is mediated by PCYT2, which can damage the glycan-mediated cell adhesion with the extracellular matrix, which might, therefore, lead to the metastasis of CRC [114]. Fatty acid 2-hydroxylase (FA2H) has been found to inhibit CRC migration and EMT. Mechanistically, FA 2-hydroxylation initiates a metabolic signaling cascade that inhibits CRC metastasis through the YAP transcriptional axis [115]. High mobility group box-1 (HMGB1) is a known MSC chemokine. The stemness and proliferation of MSCs can be increased by oxidized HMGB1, which increases the stemness of CRC cells and promotes metastatic potential [116]. In addition, ENO1 has been suggested to promote CRC metastasis via its crotonylation at K420 [117]. Small ubiquitin-like related modifier (SUMO) is a dynamic, multi-step conjugation and deconjugation cascade, which is catalyzed by adenosine triphosphate (ATP)-dependent enzymes. Protein SUMOylation is catalyzed by the SUMO activating enzyme E1, SUMO-specific conjugating enzyme ubiquitin-binding protein (Ubc) 9, and SUMO ligase E3. Ubiquitin-like modifier activating enzyme 2 (Uba2) is the key enzyme for the SUMO pathway. It has been reported that Uba2 induces the invasion and metastasis of CRC cells in vitro by promoting the effect on the Wnt signaling pathway [118]. Eukaryotic Elongation Factor 1 Alpha 1 demonstrated acetylation at K439, which was found to enhance migration and invasion of CRC cells. Further, the acetylation level of eEF1A1 K439 was higher in CRC patients than in healthy individuals [119]. Procollagen-lysine, 2-oxoglutarate 5-dioxygenase 2 (PLOD2) serves as a lysyl hydroxylase, which has been found to interact with USP15, which promotes CRC invasion and metastasis via activating the AKT/mTOR pathway [120] (Figure 6).

### Crosstalk between Different PTMs Contributes to CRC Metastasis

Until now, over 300 types of PTMs have been identified [121], and recent studies have indicated that different PTMs can either positively or negatively influence each other [122]. For instance, JNK induces Ser27 SIRT1 phosphorylation and promotes the progression of CRC by Snail deacetylation [123]. The methylation–phosphorylation switch regulates the stability and function of RIOK1. The K411-methylated RIOK1 is easily ubiquitinated, but the phosphorylation of RIOK1 at T410 can antagonize K411 methylation to stabilize RIOK1. It was found that RIOK1 demethylation or phosphorylation signaling enhanced CRC metastasis [124]. As an enzyme catalyzing citrullination, peptidylarginine deiminase 4 (PAD4) mediates GSK3β Arg-344 citrullination and subsequent degradation of nuclear cyclin-dependent kinase inhibitor 1 (CDKN1A), thus regulating CRC progression [125]. PAK5 phosphorylates SRSF11 at serine 287, which interferes with the Ub of the nearby K288 site and consequently protects SRSF11 from Ub-dependent degradation. Increased SRSF11 expression and alternative splicing function on HSPA12A lead to enhanced metastasis and a poor prognosis in CRC patients [126]. Zinc finger E-box–binding homeobox 1 (ZEB1) is a key transcription factor of the EMT process and is the Ubiquitinated substrate of USP10. USP10 phosphorylation at Ser236 via MEK-ERK signaling impairs its interaction with ZEB1, further protecting ZEB1 from proteasomal degradation. Therefore, the phosphorylation–deubiquitination interaction between USP10 and ZEB1 suppresses CRC metastasis [127]. Further, the dephosphorylation of ACOX1 by DUSP14 has been proven to increase β-catenin palmitoylation to promote CRC progression [128] (Figure 6). 

## 6. Therapeutic Potential of Targeting PTMs to Inhibit CRC Metastasis

Increasing evidence suggests that PTMs can not only serve as important biomarkers for early diagnosis but also provide specific targets for treatment [129]. An investigation of PTMs, which could change the conventional CRC therapies, might improve the therapeutic efficacy, including small molecule inhibitors targeting PTMs, the vaccine of PTM epitopes, and cooperation with other types of anti-cancer drugs. 

### 6.1. Drugs Targeting Phosphorylation

The anti-metastasic effect against CRC of *Astragalus membranaceus* and *Curcuma zedoaria* has been reported to induce the phosphorylation of β-catenin, leading to its degradation and the subsequent downregulation of the EMT signal [130]. Rosmarinic acid (RA) was used as an oriental medicine, which was suggested to inhibit the metastatic properties of CRC cells through the phosphorylation of AMPK [131]. CD4 helper T-lymphocytes (HTLS) and CD8 cytotoxic T-lymphocytes (CTLS) can recognize post-translationally modified antigens and normal antigens. As seventy percent of metastatic CRC samples were positive for phosphorylated vimentin, peptide-reactive CD4 T-cells recognized phosphorylated vimentin-positive tumor cells directly or indirectly, and phosphorylated vimentin could become a helper peptide vaccine to treat CRC [17]. MnTE-2-PyP can effectively inhibit the TGF-β-mediated migration and invasion of CRC cells by reducing the phosphorylation of the Smad2/3 protein induced by TGF-β in CRC cells [132]. As HER2/3 phosphorylation leads to tumor progression and resistance, a multifunctional nanoparticle formulation of afatinib and miR-139 targeting EGFR/HER/Ras/Akt/Rac1/STAT3/MAPK/EMT/Bcl-2 pathways has been found to suppress metastasis in CRC cells [133]. Because α-hederin inhibited the phosphorylation of JAK2 and STAT3, which is implicated in tumor EMT and metastasis, α-hederin could be regarded as a promising candidate for the intervention of colon cancer metastasis [134]. A novel compound, CW85319, was discovered to inhibit CRC distal metastasis by enhancing the interaction of Axin2 with GSK3β and its phosphorylation [135]. Compound K (CK) is a new ligand of Nur77, which promotes Nur77 to display resistance to Akt phosphorylation and further discouple from p63, leading to the Nur77-Akt feed-forward loop disruption and the regulation of CRC metastasis [136]. In addition, Buddlejasaponin IV (BS-IV) acts as an anti-metastatic agent by reducing FAK and Akt phosphorylation levels, which inhibits the lung metastases of colon cancer cells [137]. 

### 6.2. Drugs Targeting Glycosylation

Intestinal microecology has been widely reported to be closely implicated in CRC. It has been found that Rhizoctonia bataticola lectin (RBL) inhibits the angiogenesis and metastasis of CRC cells. The underlying mechanism is that hypermannose N-glycans, expressed in large quantities in human colon cancer cells, and Lectins recognize cancer-associated antigens and subsequently trigger apoptosis. Furthermore, RBL has the ability to recognize tumor-specific glycans from both metastatic CRC and primary CRC and uses different binding [138]. The Sialyl–Thomsen-nouveau (STn) antigen is a tumor-associated carbohydrate antigen that is the result of incomplete O-glycosylation. An anti-STn monoclonal antibody named L2A5 reacts with tumor-associated O-glycosylated proteins and might become a novel therapy strategy for CRC [139]. 

### 6.3. Drugs Targeting Ubiquitination

The E3 ubiquitin-ligase Hakai has been suggested to be associated with the degradation of E-cadherin. Unlike other inhibitors, the novel specific inhibitors of Hakai can directly affect EMT. Hakin-1, inhibiting Hakai-dependent ubiquitination of E-cadherin, subsequently affects the EMT process of CRC [140].

## 7. Conclusions and Future Perspectives

The ability of CRC cells to metastasize is highly dependent on cellular properties such as EMT status, stem cell plasticity, genetics, epigenetics, chromosome instability, and metabolic adaption [141]. Various PTMs regulate diverse protein functions of stability, localization, or interaction, which control different biological processes, including the metastasis of CRC. Currently, common PTMs such as phosphorylation, ubiquitination, glycosylation, and acetylation have been widely investigated and closely linked to multiple aspects of CRC metastasis [142]. Future studies might discover more novel PTMs, such as methylation, SUMOylation, palmitoylation, and lactylation, and their impact on CRC metastasis. Until now, a variety of PTMs have been identified, and an increasing number of studies indicate that a complex network of crosstalk between different PTMs contributes to CRC metastasis. The cooperation of different PTMs might generate new targets and strategies for the treatment of CRC metastases. In addition, the combination of gene signatures, mass spectrometry analysis, and multiple new metabolomics and proteomics might be a better solution to unravel the underlying mechanism of CRC metastasis [143]. 

Increasing evidence suggests that PTMs might serve as potential targets for the prevention and treatment of CRC metastases [144]. Investigation of PTMs, which can change the conventional CRC therapies, might improve therapeutic efficacy, including small molecule inhibitors targeting PTMs, the vaccine of PTMs epitopes, and cooperation with other types of anti-cancer drugs [145]. With the development of nanotechnology, the interdisciplinary cooperation between medicine and engineering is deepening, and new drug delivery methods are constantly innovating to achieve better therapeutic effects. In addition to new artificial drugs, the use of the body’s own regulatory mechanisms to regulate PTMs is also a hot prospect, such as signaling proteins, enzymes, metabolites, lipids, long non-coding RNAs, microRNAs [146], etc. For example, it has been reported that exosomes that come from BMSCs could inhibit CRC migration and invasion by regulating PI3K/Akt activation [147]. Along with the rapid development of mass spectrometry [148], proteomics, single-cell sequencing, and spatial genomics, more novel pharmacological targets of PTMs will be identified, which would greatly benefit the early prevention and clinical treatment of CRC metastasis.

## Figures and Tables

**Table 1:** E3 Ubiquitin ligases that are associated with colorectal cancer metastasis.

E3	Substrate	Inhibit/Promote Metastasis	Cellular Function	Molecular Pathway	References
NEDD4	F0XA1	promote	EMT, migration, invasion Invasion	N/A	[62]
NEDD4	P21	promote	EMT, migration, invasion	N/A	[63]
HERC3	EIF5A2	inhibit	metastasis	TGF-/smad2/3	[64]
RNF6	STAT3	promote	metastasis	JAK-STAT3	[65]
RNF6	TLE3	promote	EMT	WNT/β-catenin	[66]
RNF126	P53	promote	migration	P21/Rb	[67]
RNF43	Fzd	inhibit	stemness	WNT	[68]
pVHL	HIF-1α	inhibit	EMT, metastasis	N/A	[69]
TRIM65	ARHGAP35	promote	Migration, metastasis	N/A	[70]
TRIM47	SMAD4	promote	invasion	CCL15-CCR1	[71]
TRIM28	CARM 1	inhibit	EMT	WNT/β-catenin	[72]
TRIM16	snail	inhibit	EMT, migration, invasion	N/A	[73]
TRAF6	N/A	promote	Migration, invasion	NF-κB- VEGF-C	[74]
TRAF6	LC3B	inhibit	EMT, metastasis	WNT/β-catenin	[75]
RBBP6	N/A	promote	EMT, metastasis	NF-κB pathway	[76]
Fbx8	GSTP1	Inhibit	Invasion	N/A	[77]
FBXW7	N/A	promote	metastasis	N/A	[78]
SMURF2	RhoA	Inhibit	migration and invasion	N/A	[79]
SMURF2	EpCAM	Inhibit	stemness	N/A	[80]

The meaning of abbreviation N/A is Not Available.
